# Relationship between ultraviolet index (UVI) and first-, second- and third-degree sunburn using the Probit methodology

**DOI:** 10.1038/s41598-018-36850-x

**Published:** 2019-01-24

**Authors:** J. F. Sánchez-Pérez, D. Vicente-Agullo, M. Barberá, E. Castro-Rodríguez, M. Cánovas

**Affiliations:** 1 Department of Applied Physics, Universidad Politécnica de Cartagena (UPCT), Cartagena, Spain; 2 General Directorate of Public Health, Environmental Health Section, Comunitat Valenciana, Spain; 3 Metallurgical and Mining Engineering Department, Universidad Católica del Norte, Antofagasta, Chile

## Abstract

In this paper, a relation between the ultraviolet index (UVI) as a Sun exposure time and its effects in the form of burns according to the skin type has been elaborated. Moreover, we present a new expression that relates the intensity of solar radiation and the UVI, as well as expressions to obtain the percentage of population affected both by first and second degree lllsunburn for every skin-type. The results have been adjusted and validated through experimental results taken from the bibliography. Finally, this paper presents a table where the population can easily interpret the UVI values and calculate the maximum time one can be exposed to solar radiation without getting sunburn. In addition, this article aims to raise awareness of the potential harm caused by solar radiation by indicating the percentage of population affected by different types of sunburn depending on skin-type. Moreover, ultraviolet exposure to sunlight could not just result in sunburn, but also have long-term effects on eyes, or even cause immune system disorders or melanoma. Therefore, managing risk perception with this useful table could familiarize the population with actual harm prevention.

## Introduction

Sunburn is the reddening of the skin that occurs after having been overexposed to the Sun radiation or other types of ultraviolet light, because melanin is not able to protect the skin when there is a high degree of exposure to the Sun (or a source of ultraviolet light). Melanin is the coloration (pigment) that protects the skin. Sunburn on a person with very fair skin can occur in less than 15 minutes of exposure to the Sun at noon, while a person with darker skin can tolerate the same exposure for hours^1^. Due to these differences, the World Health Organization (WHO) divided the type of skin according to its melanin levels and, therefore, its resistance to sunburn^1^. One of the main effects of solar radiation is first and, less frequently, second-degree sunburn^1^. To establish a simple methodology to inform the population of the intensity of solar radiation, the ultraviolet index (UVI) was established, a simple number that informs of the dangerousness of the sunlight as a function of the Earth’s relative position to the Sun, altitude, cloudiness, etc^2^. Sometimes, this index is difficult to interpret by the general population, since they do not perceive the danger that it could cause to their skin. Thus, in this article we propose to establish a direct relationship between this index and the different degrees of sunburn as a function of the exposure time and skin type, providing the population with a greater perception of the danger posed by solar radiation. According to WHO guidelines, UV exposure should be controlled. A moderate daily degree of this radiation is essential for the synthesis of Vitamin D through skin, liver and kidney pathway. This process is necessary for the regulation of calcium homeostasis and bone metabolism, providing a proper bone health. In addition, more recently other functions have been described, for example its regulatory role in immunity in humans, its effect on inflammation (attenuating inflammation), and its benefits for cardiovascular health and cancer. As for the latter, an insufficient exposure to this radiation can lead to deficiency or insufficiency in serum, and this may affect the immune and cardiovascular system, causing neurocognitive dysfunction, different types of cancer and diabetes mellitus inter alia, as cell composition in all these systems has Vitamin D receptors. Therefore, a well-balanced UVI exposure is mandatory for protecting skin, other organs, as well as for a proper bone health^3–7^. However, overexposure to sunlight can lead to unwanted effects such as redness, pain and swelling of the skin, as well as burns. Although the global burden of disease due to UVI exposure may be considered as low for some researchers^8,9^, managing a public awareness tool would encourage Sun-protective behavior by decision making of risk perception. It would not only help to prevent sunburn as an immediate effect but also further conditions such as solar keratosis, reactivation of herpes labialis, squamous cell or basal cell carcinoma, cutaneous melanoma or cortical cataract. The UVI is a relevant communicating tool in public health, as it may increase awareness about the risks of excessive exposure to UV radiation and warn about the need to adopt preventive measures^3^.

## Radiation Intensity and Dose

The ultraviolet index, UVI, formulated using the spectrum of erythematic action induced by UV radiation on human skin, of the CIE (Commission Internationale de l’Eclairage, acronym in French), is a simple index that informs the population of the risk of solar radiation^2^.

1 $${\rm{UVI}}={{\rm{k}}}_{{\rm{er}}}{\int }_{250\,{\rm{nm}}}^{400\,{\rm{nm}}}{\rm{I}}({\rm{\lambda }}){\rm{\varepsilon }}({\rm{\lambda }})d{\rm{\lambda }}$$

where I(λ) is the solar spectral irradiance in W/(m^2^·nm) and k_er_ a constant equal to 40 m^2^/W. The erythema reference action spectrum coefficient, ε(λ), values are given by Madronich *et al*.^5^.

$$\begin{array}{ll}\lambda  < 298\,{\rm{nm}} & {\rm{\varepsilon }}({\rm{\lambda }})=1\\ 298\,{\rm{nm}}\le {\rm{\lambda }} < 328\,{\rm{nm}} & {\rm{\varepsilon }}({\rm{\lambda }})={10}^{0.0940\cdot (298-1000{\rm{\lambda }})}\\ 328\,{\rm{nm}}\le {\rm{\lambda }}\le 400\,{\rm{nm}} & {\rm{\varepsilon }}({\rm{\lambda }})={10}^{0.0150\cdot (139-1000{\rm{\lambda }})}\\ \lambda  > 400\,{\rm{nm}} & {\rm{\varepsilon }}({\rm{\lambda }})=0\end{array}$$

As shown in Table 1, the ultraviolet index, UVI, a dimensionless number, can vary between 0 and 16 and it has been divided into five ranges of danger for the population according to exposure^2,10^.

**Table 1:** UV radiation exposure categories.

**UVI range**	≤2	3–5	6–7	8–10	≥11
**Exposure category**	Low	Moderate	High	Very high	Extreme

Numerous organizations inform the population about the daily values of this index. The prediction provides the maximum UV index value that matches the UV index at noon for clear skies. On cloudy days, the value of the UV index would be lower. In addition, it also sets up information of hourly values. In Spain, it can be consulted in the Agencia Estatal de Meteorología (AEMET)^11^. You can consult the UV index of any country through the website of the Finnish Meteorological Institute^12^.

The intensity of ultraviolet radiation B, I_UVB_, in the range of 280–315 nm wavelength, and measure in W/m^2^, is the main component of the solution to the integral given in expression (1), since the values for this range of the erythema reference action spectrum coefficient ε(λ) nearly occupy the entire wavelength of the ultraviolet index, UVI. Thus, some authors like MacKenzie *et al*.^13^ established the following relationship between ultraviolet index, UVI, and intensity of ultraviolet radiation B, I_UVB_:

2 $${{\rm{I}}}_{{\rm{UVB}}}=18.9\cdot {\rm{UVI}}\,{({\rm{W}}/{\rm{m}}}^{2})$$

As it is known, solar radiation can cause skin burns after prolonged exposure. Many authors relate skin temperature with the burn-degree, which in turn depends both on exposure time and on radiant intensity. Thus, these authors, Buettner or Hardee and Lee^14,15^, relate exposure time with skin temperature at different depths. However, temperature is not the correct parameter to establish the damage level, but a parameter that relates damage to exposure time (t_e_, s) and UV radiation intensity (I, W/m^2^) as it is the “UV radiation dose”. Equation (3) shows the most recognized expression for the dose (D, (W/m^2^)^4/3^ s)^16,17^.

3 $${\rm{D}}={{\rm{I}}}^{4/3}{{\rm{t}}}_{{\rm{e}}}$$

## Skin Types

Not all skin types react in the same way to Sun exposure. In this regard, the World Health Organization (WHO)^2^ has classified phototypes or skin types into six categories according to their tolerance to solar radiation, as described in the Table 2, so that skin type I is the most sensitive and type VI is the most tolerant to UV radiation.

**Table 2:** Classification of skin types^2^.

Skin type classification	Burns probability in the sun	Tans after having been in the sun
I. Melano-compromised	Always	Seldom
II. Melano-compromised	Usually	Sometimes
III. Melano-competent	Sometimes	Usually
IV. Melano-competent	Seldom	Always
V. Melano-protected	Naturally brown skin	
VI. Melano-protected	Naturally black skin	

One of the goals of public health is to protect the most vulnerable population groups. Given that the finding that more than 90% of non-melanoma skin cancers occur in people of skin type I and II, protection messages should be focused on people of these skin types, who tend to get burnt easily. In addition, special mention should be made of children, who are more sensitive to UV radiation. Therefore, it is understood that the development of equivalence between the UVI and the percentage of population affected by different degree sunburn will promote awareness of solar radiation among that population. Although dark-skinned people have a lower risk of cancer, they can suffer from other harmful consequences, such as burns to the eyes, solar retinopathy, photo conjunctivitis or photo keratosis.

Thus, to advise on the dangers of solar radiation, several cultural and climate differences must be taken into account, namely, the population’s perception of UV radiation risks and their education^2^. Therefore, we believe that the relation between UVI and the percentage of population affected by different degree burns is a useful tool for raising awareness of solar radiation harm among the population.

## Effects of Solar Radiation on Human Population

The generation of skin sunburn, in its different degrees, is a significant effect of UV radiation, which generates free radicals leading to inflammation. The seriousness varies depending on the proportion of burned skin and the depth of the most severe burn.

The effects caused by UV radiation in humans can be divided into two types. First, physiological effects: increased heart rate, increased transpiration or body temperature. Second, pathological effects, which are more serious and include skin burns. These burns are divided into three categories depending on the seriousness of the damage: first degree, second degree and third degree burns.

First-degree sunburn produces redness, swelling, pain and superficial damage. These burns only affect the skin surface (epidermis) and cause negligible tissue damage. This kind of burn does not require medical attention, since they do not end up in blisters and their effects might be reversible in a period of 1 or 2 days.

The approximate value of the dose established for the appearance of first-degree sunburn is 115 (kW/m^2^)^4/3^s, given by Sanchez-Perez *et al*.^18^ and adjusted from the values given by Buettner and Bagster and Pitblado^19,20^.

**Figure 1:**
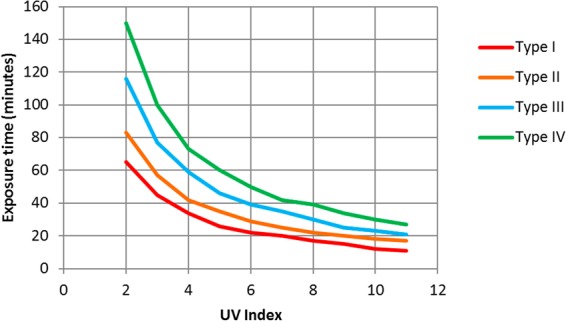
Relationship between exposure time for the appearance of first-degree sunburn and UV index for several skin types and 1 Minimal Erythema Dose (MED) according to DIN-5050^21,22^ for clear sky days. Built from the information given by^10,21–23^.

In several studies on the ultraviolet index^10,21–23^, UVI, an association between skin type and maximum exposure time (in minutes) in which first-degree sunburn would appear is established (Fig. 1).

**Figure 2:**
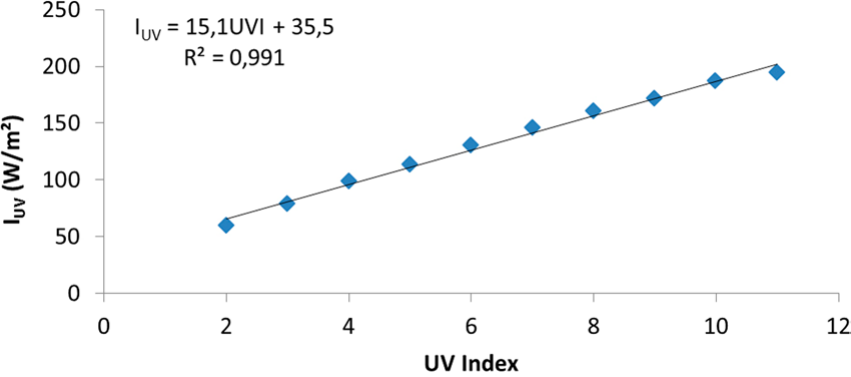
Relationship between UVI and intensity of ultraviolet radiation for the beginning of first-degree sunburn in skin type II.

For skin type II and a value of 9 for UV index, using expression (2), the corresponding dose value is 113 (kW/m^2^)^4/3^s, next to value 115 (kW/m^2^)^4/3^s, given by Sanchez-Perez *et al*.^18^ and adjusted from the experimental values given by Buettner and Bagster and Pitblado^19,20^ for the beginning of first-degree burns. However, if we use expression (2) given by MacKenzie *et al*.^13^, for the dose calculation for lower values of UV index we do not obtain an approximate value of 115 (kW/m^2^)^4/3^s. Thus, adjusting the curve for a type II to an approximate dose value of 115 (kW/m^2^)^4/3^s, Table 3, and using equation (3), we get a new expression that relates the ultraviolet index, UVI, and the intensity of ultraviolet radiation, I_UV_, (Fig. 2).

**Table 3:** Relationship between UVI and exposure time for the appearance of first-degree sunburn in skin type II according to DIN-5050. Built from the information given by^10,21–23^.

UV Index	Time (min)
2	83
3	57
4	42
5	35
6	29
7	25
8	22
9	20
10	18
11	17

The proposal for an expression that relates the ultraviolet index, UVI, and the intensity of ultraviolet radiation, I_UV_, is given by:

4 $${{\rm{I}}}_{{\rm{UV}}}=15.1\cdot {\rm{UVI}}+35.5({\rm{W}}/{{\rm{m}}}^{{\rm{2}}})$$

This expression, which mainly includes ultraviolet B radiation, covers the wavelength from 250 nm to 400 nm, so it is closer to the UVI calculation given by expression (1) than the expression proposed by MacKenzie *et al*.^13^, since it encompasses the variability of the erythema reference action spectrum coefficient ε(λ).

Considering that each skin type has a different dose for the beginning of first-degree sunburn (Fig. 1), a dose can be established for each type using expressions (3) and (4), and the values of the UV index and exposure times in Fig. 1.

The value for skin type II coincides with the value of 115 (kW/m^2^)^4/3^s given by Sanchez-Perez^18^ validating the use of these expressions for solar radiation. For the rest of skin types, new dose levels are established for the initiation of first-degree burns, (Table 4).

**Table 4:** Beginning dose of first-degree sunburn for each skin type.

Skin type classification	Dose (kW/m^2^)^4/3^s
I	84.9 ± 1.67
II	115 ± 1.80
III	143 ± 4.57
IV	195 ± 4.19

Note: The 95% confidence interval has been included in each dose.

Second-degree burns affect the epidermis (outer-layer) and the dermis (underlying layer of the skin), causing blisters, pain, swelling and redness. Contrary to first-degree burns, in this kind of burns a medical treatment is required to heal the damaged area^24^. Using data collected by Stoll and Greene^25^ and Metha *et al*.^26^, a value of dose is established for the beginning of second-degree burns of 250 (kW/m^2^)^4/3^s.

Third-degree burns affect the deep hypodermis, dermis and epidermis, causing skin carbonization or a translucent white color, with coagulated and visible vessels under the skin surface. The healing of these burns occurs slowly due to tissue destruction. Consequently, people with these types of burns are more vulnerable to infections^24^.

## Probit Equations to Determine Solar Effects

Probit equations^27^ relate the percentage of population affected by a certain damage level (e.g., first-, second- or third-degree sunburn) and solar radiation. The dimensionless number Probit (Y) and its interpretation allow calculating the population percentage affected by this radiation, Sánchez-Pérez *et al*.^18^ and TNO (1989)^16^:

5 $$\mathrm{First} \mbox{-} \mathrm{degree}\,{\rm{burns}}\,{\rm{Y}}=-\,11.7+6.95\,\mathrm{log}({\rm{D}})$$

6 $$\mathrm{Second} \mbox{-} \mathrm{degree}\,{\rm{burns}}\,{\rm{Y}}=-\,13.9+6.95\,\mathrm{log}({\rm{D}})$$

7 $$\mathrm{Third} \mbox{-} \mathrm{degree}\,{\rm{burns}}\,{\rm{Y}}=-\,36.4+2.56{\rm{In}}({\rm{D}})$$

In the case of first-degree sunburn, an adjustment must be made depending on the skin type. Given that the value of population affected by first-degree sunburn is 1%, Y = 2.67, and using the values given in Table 4, for each skin type the following expressions are proposed:

8 $$\begin{array}{cc}\mathrm{First} \mbox{-} \mathrm{degree}\,{\rm{burns}}\,{\rm{Probit}}\,{\rm{equation}}\,{\rm{for}}\,{\rm{skin}}\,{\rm{type}}\,{\rm{I}} & {\rm{Y}}=-\,10.8+6.95\,\mathrm{log}({\rm{D}})\end{array}$$

9 $$\begin{array}{cc}\mathrm{First} \mbox{-} \mathrm{degree}\,{\rm{burns}}\,{\rm{Probit}}\,{\rm{equation}}\,{\rm{for}}\,{\rm{skin}}\,{\rm{type}}\,{\rm{II}} & {\rm{Y}}=-\,11.7+6.95\,\mathrm{log}({\rm{D}})\end{array}$$

10 $$\begin{array}{cc}\mathrm{First} \mbox{-} \mathrm{degree}\,{\rm{burns}}\,{\rm{Probit}}\,{\rm{equation}}\,{\rm{for}}\,{\rm{skin}}\,{\rm{type}}\,{\rm{III}} & {\rm{Y}}=-\,12.3+6.95\,\mathrm{log}({\rm{D}})\end{array}$$

11 $$\begin{array}{cc}\mathrm{First} \mbox{-} \mathrm{degree}\,{\rm{burns}}\,{\rm{Probit}}\,{\rm{equation}}\,{\rm{for}}\,{\rm{skin}}\,{\rm{type}}\,{\rm{IV}} & {\rm{Y}}=-\,13.2+6.95\,\mathrm{log}({\rm{D}})\end{array}$$

The expressions given by Sánchez-Pérez *et al*.^18^ and TNO^16^ are maintained for the calculation of the rest of the affected population percentage, that is, second- and third-degree burns. For the interpretation of the Probit value, the Probit tables can be used, which establish a relation between Probit number and the percentage of population affected^16,18,27–32^ or the following equation^27–32^:

12 $${\rm{R}}=-\,3.25{{\rm{Y}}}^{3}+48.8{{\rm{Y}}}^{2}-\,207{\rm{Y}}+270$$

where R is the percentage of affected population and valid for a percentage range between 5 and 95%, that is, valid for Probit values between 3.36 and 6.64.

The interpretation of the results obtained from the previous equations is done through the corrected tables for doses and Probit given by Sanchez-Perez *et al*.^18^, thus determining the percentage of people affected by the different degrees of burns.

## Relationship Between UVI and Percentage of Population Affected by Each Type of Burn

Following the proceeding established by Sanchez-Perez *et al*.^18^ for the calculation of percentages of affected population using the Probit equations and expressions (2) and (3), the following table (Table 5) is established, which relates the percentage of population affected for each UVI, exposure time and skin type.

**Table 5:** Relationship between UVI and percentage of population affected by each burn type. Different expressions for each type of skin in first-degree burns.

UVI	Exposition time (minutes)	Dose (kW/m^2^)^4/3^s	Skin type	First-degree burns (%)	Second-degree burns (%)	Third-degree burns (%)
6	25	95.12	I	2	0	0
			II	0	0	0
			III	0	0	0
			IV	0	0	0
	30	114.14	I	6	0	0
			II	1	0	0
			III	0	0	0
			IV	0	0	0
	35	133.16	I	16	0	0
			II	3	0	0
			III	0	0	0
			IV	0	0	0
	40	152.18	I	28	0	0
			II	7	0	0
			III	1	0	0
			IV	0	0	0
	45	171.21	I	40	0	0
			II	13	0	0
			III	3	0	0
			IV	0	0	0
	50	190.23	I	53	0	0
			II	21	0	0
			III	7	0	0
			IV	0	0	0
	55	209.25	I	63	0	0
			II	31	0	0
			III	11	0	0
			IV	1	0	0
	60	228.28	I	72	0	0
			II	41	0	0
			III	18	0	0
			IV	3	0	0
7	20	88.48	I	1	0	0
			II	0	0	0
			III	0	0	0
			IV	0	0	0
	25	110.60	I	6	0	0
			II	0	0	0
			III	0	0	0
			IV	0	0	0
	30	132.72	I	15	0	0
			II	2	0	0
			III	0	0	0
			IV	0	0	0
	35	154.84	I	30	0	0
			II	7	0	0
			III	1	0	0
			IV	0	0	0
	40	176.96	I	44	0	0
			II	15	0	0
			III	4	0	0
			IV	0	0	0
	45	199.08	I	58	0	0
			II	26	0	0
			III	9	0	0
			IV	1	0	0
	50	221.20	I	69	0	0
			II	37	0	0
			III	16	0	0
			IV	2	0	0
	55	243.32	I	78	1	0
			II	47	1	0
			III	23	1	0
			IV	4	1	0
	60	265.44	I	84	2	0
			II	55	2	0
			III	31	2	0
			IV	6	2	0
8	20	101.32	I	3	0	0
			II	0	0	0
			III	0	0	0
			IV	0	0	0
	25	126.65	I	12	0	0
			II	2	0	0
			III	0	0	0
			IV	0	0	0
	30	151.98	I	28	0	0
			II	7	0	0
			III	1	0	0
			IV	0	0	0
	35	177.31	I	45	0	0
			II	16	0	0
			III	4	0	0
			IV	0	0	0
	40	202.64	I	60	0	0
			II	28	0	0
			III	10	0	0
			IV	1	0	0
	45	227.97	I	72	0	0
			II	40	0	0
			III	18	0	0
			IV	3	0	0
	50	253.30	I	81	1	0
			II	51	1	0
			III	27	1	0
			IV	5	1	0
	55	278.63	I	87	3	0
			II	60	3	0
			III	35	3	0
			IV	7	3	0
	60	303.96	I	88	5	0
			II	67	5	0
			III	43	5	0
			IV	11	5	0
9	20	114.58	I	7	0	0
			II	1	0	0
			III	0	0	0
			IV	0	0	0
	25	143.23	I	22	0	0
			II	4	0	0
			III	1	0	0
			IV	0	0	0
	30	171.87	I	41	0	0
			II	13	0	0
			III	4	0	0
			IV	0	0	0
	35	200.52	I	59	0	0
			II	27	0	0
			III	9	0	0
			IV	1	0	0
	40	229.16	I	73	0	0
			II	41	0	0
			III	19	0	0
			IV	3	0	0
	45	257.81	I	83	1	0
			II	53	1	0
			III	29	1	0
			IV	6	1	0
	50	286.45	I	88	3	0
			II	63	3	0
			III	38	3	0
			IV	9	3	0
	55	315.10	I	88	6	0
			II	70	6	0
			III	46	6	0
			IV	14	6	0
	60	343.74	I	86	11	0
			II	72	11	0
			III	51	11	0
			IV	16	11	0
10	20	128.24	I	13	0	0
			II	2	0	0
			III	0	0	0
			IV	0	0	0
	25	160.30	I	34	0	0
			II	9	0	0
			III	2	0	0
			IV	0	0	0
	30	192.36	I	54	0	0
			II	23	0	0
			III	7	0	0
			IV	0	0	0
	35	224.42	I	71	0	0
			II	39	0	0
			III	17	0	0
			IV	2	0	0
	40	256.48	I	82	1	0
			II	53	1	0
			III	29	1	0
			IV	6	1	0
	45	288.53	I	87	4	0
			II	63	4	0
			III	38	4	0
			IV	8	4	0
	50	320.59	I	88	7	0
			II	70	7	0
			III	47	7	0
			IV	14	7	0
	55	352.65	I	85	12	0
			II	74	12	0
			III	53	12	0
			IV	19	12	0
	60	384.71	I	79	19	0
			II	73	19	0
			III	56	19	0
			IV	21	19	0
11	20	142.27	I	22	0	0
			II	4	0	0
			III	0	0	0
			IV	0	0	0
	25	177.83	I	45	0	0
			II	16	0	0
			III	4	0	0
			IV	0	0	0
	30	213.40	I	65	0	0
			II	33	0	0
			III	13	0	0
			IV	2	0	0
	35	248.97	I	81	0	0
			II	50	0	0
			III	27	0	0
			IV	5	0	0
	40	284.55	I	87	3	0
			II	62	3	0
			III	38	3	0
			IV	9	3	0
	45	320.10	I	88	7	0
			II	70	7	0
			III	47	7	0
			IV	14	7	0
	50	355.67	I	84	13	0
			II	73	13	0
			III	52	13	0
			IV	19	13	0
	55	391.24	I	79	19	0
			II	72	19	0
			III	56	19	0
			IV	23	19	0
	60	426.80	I	70.4	29	0
			II	66	29	0
			III	54	29	0
			IV	23	29	0

From Table 5 it can be concluded that the results for the first-degree sunburn are in accordance with reality. However, for the second degree there is a high percentage of affected population for types of skin, which are more resistant to burns, such as Type III and IV. This is because only the values given by Stoll and Greene^25^ and Metha *et al*.^26^ for the initiation of second-degree burns are irrespective of skin type. In order to distinguish the start of second degree burns for each skin type, experimental results obtained by Stoll and Greene^25^ and Metha *et al*.^26^, who do not distinguish the type of skin, are adjusted using the experimental data of first degree burns, which do distinguish skin type.

Thus, it is proposed that set the start of second-degree burns up 250 (kW/m^2^)^4/3^s^18^, for skin type II, and the start of second-degree burns for the rest of skins type is adjusted with the data of each skin type of first-degree burns. Therefore, the values of Table 6 can be obtained.

**Table 6:** Beginning dose of second-degree burns for each skin type.

Skin type classification	First-degree burns dose (kW/m^2^)^4/3^s	Second-degree burns dose (kW/m^2^)^4/3^s
I	84.9 ± 1.67	220 ± 1.67
II	115 ± 1.80	250 ± 1.80
III	143 ± 4.57	279 ± 4.57
IV	195 ± 4.19	330 ± 4.19

Note: The 95% confidence interval has been included in each dose.

These values are in the range of those given by different authors for the initiation of second-degree burns, 236–390 (kW/m^2^)^4/3^s, without specifying the skin type^16,18,20,33^. Thus, the following expressions of Probit equations can be proposed adjusting the value of 1% population affected by second-degree burns, Y = 2.67, for each type of skin using the values given in Table 6.

13 $$\begin{array}{cc}\mathrm{Second} \mbox{-} \mathrm{degree}\,{\rm{burns}}\,{\rm{Probit}}\,{\rm{equation}}\,{\rm{for}}\,{\rm{skin}}\,{\rm{type}}\,{\rm{I}} & {\rm{Y}}=-\,13.6+6.95\,\mathrm{log}({\rm{D}})\end{array}$$

14 $$\begin{array}{cc}\mathrm{Second} \mbox{-} \mathrm{degree}\,{\rm{burns}}\,{\rm{Probit}}\,{\rm{equation}}\,{\rm{for}}\,{\rm{skin}}\,{\rm{type}}\,{\rm{II}} & {\rm{Y}}=-\,13.9+6.95\,\mathrm{log}({\rm{D}})\end{array}$$

15 $$\begin{array}{cc}\mathrm{Second} \mbox{-} \mathrm{degree}\,{\rm{burns}}\,{\rm{Probit}}\,{\rm{equation}}\,{\rm{for}}\,{\rm{skin}}\,{\rm{type}}\,{\rm{III}} & {\rm{Y}}=-\,14.3+6.95\,\mathrm{log}({\rm{D}})\end{array}$$

16 $$\begin{array}{cc}\mathrm{Second} \mbox{-} \mathrm{degree}\,{\rm{burns}}\,{\rm{Probit}}\,{\rm{equation}}\,{\rm{for}}\,{\rm{skin}}\,{\rm{type}}\,{\rm{IV}} & {\rm{Y}}=-\,14.8+6.95\,\mathrm{log}({\rm{D}})\end{array}$$

Therefore, using the above expressions, Table 5 can be rewritten in the following table (Table 7).

**Table 7:** Relationship between UVI and percentage of population affected by each burn type. Different expressions for each skin type in first- and second-degree burns.

UVI	Exposition time (minutes)	Dose (kW/m^2^)^4/3^s	Skin type	First-degree burns (%)	Second-degree burns (%)	Third-degree burns (%)
6	25	95.12	I	2	0	0
			II	0	0	0
			III	0	0	0
			IV	0	0	0
	30	114.14	I	6	0	0
			II	1	0	0
			III	0	0	0
			IV	0	0	0
	35	133.16	I	16	0	0
			II	3	0	0
			III	0	0	0
			IV	0	0	0
	40	152.18	I	28	0	0
			II	7	0	0
			III	1	0	0
			IV	0	0	0
	45	171.21	I	40	0	0
			II	13	0	0
			III	3	0	0
			IV	0	0	0
	50	190.23	I	53	0	0
			II	21	0	0
			III	7	0	0
			IV	0	0	0
	55	209.25	I	63	0	0
			II	31	0	0
			III	11	0	0
			IV	1	0	0
	60	228.28	I	71	1	0
			II	41	0	0
			III	18	0	0
			IV	3	0	0
7	20	88.48	I	1	0	0
			II	0	0	0
			III	0	0	0
			IV	0	0	0
	25	110.60	I	6	0	0
			II	0	0	0
			III	0	0	0
			IV	0	0	0
	30	132.72	I	15	0	0
			II	2	0	0
			III	0	0	0
			IV	0	0	0
	35	154.84	I	30	0	0
			II	7	0	0
			III	1	0	0
			IV	0	0	0
	40	176.96	I	44	0	0
			II	15	0	0
			III	4	0	0
			IV	0	0	0
	45	199.08	I	58	0	0
			II	26	0	0
			III	9	0	0
			IV	1	0	0
	50	221.20	I	68	1	0
			II	37	0	0
			III	16	0	0
			IV	2	0	0
	55	243.32	I	77	2	0
			II	47	1	0
			III	24	0	0
			IV	5	0	0
	60	265.44	I	83	3	0
			II	55	2	0
			III	33	0	0
			IV	8	0	0
8	20	101.32	I	3	0	0
			II	0	0	0
			III	0	0	0
			IV	0	0	0
	25	126.65	I	12	0	0
			II	2	0	0
			III	0	0	0
			IV	0	0	0
	30	151.98	I	28	0	0
			II	7	0	0
			III	1	0	0
			IV	0	0	0
	35	177.31	I	45	0	0
			II	16	0	0
			III	4	0	0
			IV	0	0	0
	40	202.64	I	60	0	0
			II	28	0	0
			III	10	0	0
			IV	1	0	0
	45	227.97	I	71	1	0
			II	40	0	0
			III	18	0	0
			IV	3	0	0
	50	253.30	I	80	2	0
			II	51	1	0
			III	28	0	0
			IV	6	0	0
	55	278.63	I	85	5	0
			II	60	3	0
			III	37	1	0
			IV	10	0	0
	60	303.96	I	84	9	0
			II	67	5	0
			III	46	2	0
			IV	16	0	0
9	20	114.58	I	7	0	0
			II	1	0	0
			III	0	0	0
			IV	0	0	0
	25	143.23	I	22	0	0
			II	4	0	0
			III	1	0	0
			IV	0	0	0
	30	171.87	I	41	0	0
			II	13	0	0
			III	4	0	0
			IV	0	0	0
	35	200.52	I	59	0	0
			II	27	0	0
			III	9	0	0
			IV	1	0	0
	40	229.16	I	72	1	0
			II	41	0	0
			III	19	0	0
			IV	3	0	0
	45	257.81	I	81	3	0
			II	53	1	0
			III	30	0	0
			IV	7	0	0
	50	286.45	I	85	6	0
			II	63	3	0
			III	40	1	0
			IV	11	0	0
	55	315.10	I	84	10	0
			II	70	6	0
			III	50	2	0
			IV	20	0	0
	60	343.74	I	81	16	0
			II	72	11	0
			III	58	4	0
			IV	26	1	0
10	20	128.24	I	13	0	0
			II	2	0	0
			III	0	0	0
			IV	0	0	0
	25	160.30	I	34	0	0
			II	9	0	0
			III	2	0	0
			IV	0	0	0
	30	192.36	I	54	0	0
			II	23	0	0
			III	7	0	0
			IV	0	0	0
	35	224.42	I	70	1	0
			II	39	0	0
			III	17	0	0
			IV	2	0	0
	40	256.48	I	80	3	0
			II	53	1	0
			III	30	0	0
			IV	7	0	0
	45	288.53	I	85	6	0
			II	63	4	0
			III	41	1	0
			IV	12	0	0
	50	320.59	I	83	12	0
			II	70	7	0
			III	52	2	0
			IV	21	0	0
	55	352.65	I	79	18	0
			II	74	12	0
			III	60	5	0
			IV	30	1	0
	60	384.71	I	72	26	0
			II	73	19	0
			III	67	8	0
			IV	37	3	0
11	20	142.27	I	22	0	0
			II	4	0	0
			III	0	0	0
			IV	0	0	0
	25	177.83	I	45	0	0
			II	16	0	0
			III	4	0	0
			IV	0	0	0
	30	213.40	I	65	0	0
			II	33	0	0
			III	13	0	0
			IV	2	0	0
	35	248.97	I	79	2	0
			II	50	0	0
			III	27	0	0
			IV	5	0	0
	40	284.55	I	84	6	0
			II	62	3	0
			III	40	1	0
			IV	12	0	0
	45	320.10	I	84	11	0
			II	70	7	0
			III	54	2	0
			IV	21	0	0
	50	355.67	I	78	19	0
			II	73	13	0
			III	60	5	0
			IV	31	1	0
	55	391.24	I	70	28	0
			II	72	19	0
			III	66	9	0
			IV	39	3	0
	60	426.80	I	62.4	37	0
			II	66	29	0
			III	68	15	0
			IV	46	6	0

The results obtained are more in line with reality, both for first and second-degree burns, since a distinction is made between the different types of skin and its resistance to sunburn. Therefore this information is relevant for preventing further potential conditions on a sporadic occurrence (photokeratosis, photo conjunctivitis or solar retinopathy) or more severe skin affections such as melanoma or non-melanoma skin cancer (squamous cell carcinoma or basal cell carcinoma)^34^.

To facilitate its handling, Table 7 could be included in a mobile application, where users could see the probability of suffering burns through their location and the choice of their skin types, and depending on their exposure time to UV radiation.

## Conclusions

In this paper a relation between the ultraviolet index (UVI), exposure time to the Sun and its effects in the form of burns according to the skin type has been elaborated. In addition, we have presented a new expression that relates the intensity of solar radiation and the UVI. Also, we include expressions to obtain the percentage of population affected both by first and second degree burns by distinguishing the type of skin. The results have been adjusted and validated through experimental results obtained in the bibliography. Finally, as a main conclusion, this article presents a table where the population can easily interpret the UVI values and obtain the maximum exposure time they can be exposed to solar radiation without getting any sunburn depending on their skin type. In addition, this article aims to raise awareness of the danger of solar radiation by indicating the percentage of population affected by different types of burns and skins. In addition, apart from sunburn, the excessive exposure to UVR can lead to chronic sun-induced skin damage, including photoaging, skin cancer and ocular disorders. It is generally accepted that in order to be effective in raising awareness, the UVI messaging must be accurate and compelling by communicating relevant risks to population. In other words, this messaging contributes to mitigating hazards by creating risk perception around potential skin damage.

Moreover, recent changes in population habits (the practice of outdoor leisure activities and changes in clothing) along with increasing temperatures have led to longer exposure to UV radiation, with consequent risks for the population, making the adoption of preventive measures of very high importance^8^.

As a conclusion, Table 7 could be included in the mobile application where users through their location and the choice of their skin types could see the probability of suffering burns depending on the exposure time to UV radiation.

## Data Availability

The dataset used and analyzed are available from the authors.
